# 3-[(Benzo-1,3-dioxol-5-yl)amino]-4-meth­oxy­cyclo­but-3-ene-1,2-dione: polymorphism and twinning of a precursor to an anti­mycobacterial squaramide

**DOI:** 10.1107/S2053229624006211

**Published:** 2024-07-05

**Authors:** Paul R. Palme, Richard Goddard, Adrian Richter, Peter Imming, Rüdiger W. Seidel

**Affiliations:** a Institut für Pharmazie, Martin-Luther-Universität Halle-Wittenberg, Wolfgang-Langenbeck-Strasse 4, 06120 Halle (Saale), Germany; b Max-Planck-Institut für Kohlenforschung, Kaiser-Wilhelm-Platz 1, 45470 Mülheim an der Ruhr, Germany; University of Strathclyde, United Kingdom

**Keywords:** squaramide, anti­mycobacterial agent, tuberculosis, polymorphism, twinning, hydrogen bonding, crystal structure, concomitant polymorphs

## Abstract

The title compound, 3-[(benzo-1,3-dioxol-5-yl)amino]-4-meth­oxy­cyclo­but-3-ene-1,2-dione, C_12_H_9_NO_5_ (**3**), is a precursor to an anti­mycobacterial squaramide. Block-shaped crystals of a monoclinic form (**3**-I, space group *P*2_1_/*c*, *Z* = 8, *Z*′ = 2) and needle-shaped crystals of a triclinic form (**3**-II, space group *P*-1, *Z* = 4, *Z*′ = 2) were found to crystallize concomitantly. In both crystal forms, R_2_^2^(10) dimers assemble through N—H⋯O=C hydrogen bonds. These dimers are formed from crystallographically unique mol­ecules in **3**-I, but exhibit crystallographic *C*_*i*_ symmetry in **3**-II. Twinning by pseudomerohedry was encountered in the crystals of **3**-II. The conformations of **3** in the solid forms **3**-I and **3**-II are different from one another but are similar for the unique mol­ecules in each polymorph. Density functional theory (DFT) calculations on the free mol­ecule of **3** indicate that a nearly planar conformation is preferred.

## Abstract 2

3-[(Benzo-1,3-dioxol-5-yl)amino]-4-meth­oxy­cyclo­but-3-ene-1,2-dione forms concomitant polymorphs, namely, block-shaped crystals of a monoclinic form I (space group *P*2_1_/*c*, *Z* = 8, *Z*′ = 2) and needle-shaped crystals of a triclinic form II (space group *P*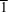, *Z* = 4, *Z*′ = 2), the latter of which exhibits twinning by pseudomerohedry.

## Introduction

Mycobacterial infections constitute a substantial threat to public health globally. These can be divided into tuberculosis (TB), infections caused by nontuberculous mycobacteria (NTM; Johansen *et al.*, 2020 ▸), and leprosy (Shyam *et al.*, 2024 ▸). According to the World Health Organization (WHO), a total of 10.6 million people worldwide fell ill with TB and an estimated number of 1.1 million deaths officially classified as caused by TB was recorded in 2022 (World Health Organization, 2023 ▸). Hard-to-cure pulmonary diseases caused by NTM are also increasingly seen (Prevots *et al.*, 2023 ▸). Drug discovery efforts are vital to fill the drug development pipelines for TB and NTM disease (Dartois & Dick, 2024 ▸). In 2012, bedaquiline was the first Federal Drug Administration (FDA)-approved novel anti-TB drug since the approval of rifampicin in 1971 (Rothstein, 2016 ▸). Bedaquiline, a di­aryl­quinolone, inhibits the proton pump of the mycobacterial ATP synthase (Andries *et al.*, 2005 ▸). Despite its success in the pharmaco­ther­apy of multidrug-resistant TB, bedaquiline exhibits some less favourable pharmacological properties, such as QTc prolongation and drug inter­actions (Deshkar & Shirure, 2022 ▸). Moreover, bedaquiline-resistant strains of *Mycobacterium tuberculosis*, the etiological agent of TB, have already emerged (Khoshnood *et al.*, 2021 ▸). Therefore, the quest for new drug candidates targeting the ATP synthase in mycobacteria is per­tinent.

**Figure 1:**
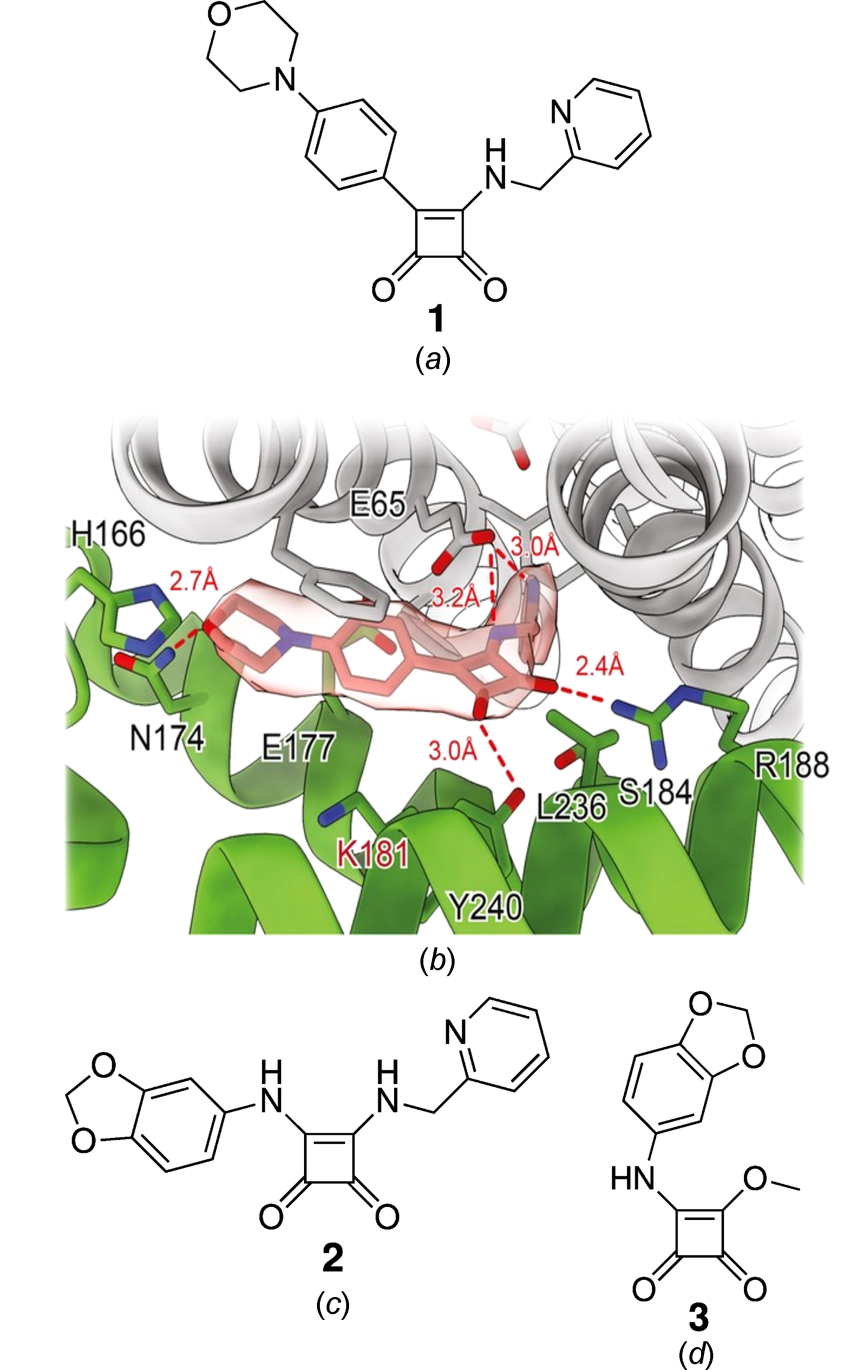
(*a*) Chemical diagram of **1** and (*b*) illustration of **1** in the complex with the *M. smegmatis* ATP synthase in the F_O_ region (PDB entry: 8g07; Courbon *et al.*, 2023 ▸). Chemical diagrams of (*c*) **2** and (*d*) its precursor **3**, the title compound. The conformation of **3** is drawn to represent that encountered in the crystal structures reported in the present work. Part (*b*) was re­pro­duced from Courbon *et al.* (2023 ▸) with permission from the publisher.

In a target-based screening of 900 000 compounds from AstraZeneca’s corporate compound collection, Tantry *et al.* (2017 ▸) discovered the compound class of squaramides as in­hibitors of the mycobacterial ATP synthesis. Structure–activity relationship (SAR) exploration and hit-to-lead optimization led to compound **1** with a mono­amino–cyclo­but-3-ene-1,2-di­one scaffold [Fig. 1 ▸(*a*)]. Compound **1** exhibited a minimum inhibitory concentration (MIC) of 0.03 µM against the reference strain *M. tuberculosis* H37Rv *in vitro* and also showed *in vivo* efficacy in a mouse model of pulmonary TB (Tantry *et al.*, 2017 ▸). Recently, Courbon *et al.* (2023 ▸) reported the structure of **1** bound to the *Mycobacterium smegmatis* ATP synthase, as determined by cryoelectron microscopy [Fig. 1 ▸(*b*)]. The results show that **1** binds to a site distinct from that of bedaquiline. Through scaffold morphing and a subsequent SAR study and optimization, Li *et al.* (2020 ▸) identified the 3,4-di­amino­cyclo­but-3-ene-1,2-dione derivative **2** [Fig. 1 ▸(*c*)], with a MIC of 0.45 µg ml^−1^ (1.4 µM) against *M. tuberculosis* H37Rv. Maintaining the 2-picolyl group proved important for activity and the introduction of a benzo-1,3-dioxole group turned out to be favourable. Compound **2** was readily obtained from amido–ester **3** [Fig. 1 ▸(*d*)] by reaction with 2-picolyl­amine.

In the course of our studies on anti­mycobacterial squaramides (Courbon *et al.*, 2023 ▸), compound **3**, the title com­pound, attracted our inter­est as a precursor to explore SARs and to optimize the potency of squaramides based on the 3,4-di­amino­cyclo­but-3-ene-1,2-dione scaffold against *M. tuberculosis* and clinically relevant NTM species. We serendipitously discovered two concomitant polymorphs of **3**, whose crystal structures we describe in the present article. Although **3** serves only as a precursor, the observed polymorphism may have broader implications in drug development (Bhatia *et al.*, 2018 ▸). As a matter of routine, we also subjected **3** to susceptibility testing against two NTM species.

## Experimental

### General

The starting materials were purchased from BLDpharm (Shanghai, China) and used as received. Methanol was distilled before use. High-performance liquid chromatography (HPLC) analysis was conducted on a Shimadzu instrument with LC-10 AD pumps and an SPD-M10A VP PDA detector, using a Polaris 5 C18-A column (5 µm, 250 mm × 4.6 mm; Agilent Technologies, Santa Clara, CA, USA) and gradient elution with water/aceto­nitrile. The flow rate was 1.2 ml min^−1^. The sample was dissolved in HPLC-grade aceto­nitrile prior to analysis. The NMR spectrum was recorded on an Agilent Technologies 400 MHz VNMRS spectrometer (ab­breviations: *s* = singlet, *bs* = broad singlet, *d* = doublet and *bd* = broad doublet).

### Synthesis and crystallization

Dimethyl squarate (1.42 g, 10 mmol) and benzo-1,3-dioxol-5-amine (1.37 g, 10 mmol) were dissolved in methanol (50 ml) and tri­ethyl­amine (2.8 ml, 20 mmol) was added. The mixture was stirred overnight at room temperature. Subsequently, the precipitate was collected by centrifugation, washed with a small amount of methanol and dried in a vacuum to yield **3** as an off-white solid (yield: 2.26 g, 9.1 mmol, 91%). HPLC purity (254 nm detection): 97.5%. ^1^H NMR (402 MHz, DMSO-*d*_6_): δ 10.59 (*s*, 1H), 6.95 (*bs*, 1H), 6.84 (*d*, 1H), 6.75 (*bd*, 1H), 5.97 (*s*, 2H), 4.33 (*s*, 3H) ppm. Block-shaped crystals of **3**-I and needle-shaped crystals of **3**-II were found when a HPLC sample of **3** in aceto­nitrile had evaporated slowly to dryness under ambient conditions.

### X-ray crystallography

After an initial independent atom model (IAM) refinement with *SHEXL2019* (Sheldrick, 2015*b* ▸), the crystal structure of **3**-I was refined with aspherical atomic form factors using *NoSpherA2* (Kleemiss *et al.*, 2021 ▸; Midgley *et al.*, 2021 ▸) in *OLEX2* (Dolomanov *et al.*, 2009 ▸). Hirshfeld-partitioned electron density was calculated in *ORCA* (Version 5.0; Neese *et al.*, 2020 ▸) using the B3LYP method (Becke, 1993 ▸; Lee *et al.*, 1988 ▸) and the def2-TZVPP basis set (Weigend & Ahlrichs, 2005 ▸). The positions and isotropic atomic displacement parameters were refined freely for all H atoms.

The crystal structure of **3**-II was refined using IAM refinement with *SHEXL2019*. The twinning was taken into account using TWIN and BASF instructions. Carbon-bound H atoms were placed in geometrically calculated positions, with aro­matic C—H = 0.95 Å, methyl­ene C—H = 0.99 Å and methyl C—H = 0.98 Å, and subsequently refined using a riding model,with *U*_iso_(H) = 1.2*U*_eq_(C) (1.5 for methyl groups). The initial torsion angles of the methyl groups were determined *via* dif­ference Fourier syntheses and subsequently refined while main­taining a tetra­hedral structure. Nitro­gen-bound H atoms were located in *F*_obs_–*F*_calc_ electron-density maps and refined semi-freely. The N1—H1 distances in both crystallographically distinct mol­ecules were restrained to be similar, with a standard uncerrtainty of 0.02 Å. The corresponding *U*_iso_(H) param­eters were refined freely.

BFDH (Bravais, Friedel, Donnay and Harker) morphologies (Bravais, 1866 ▸; Friedel, 1907 ▸) were calculated with *Mercury* (Macrae *et al.*, 2020 ▸), and packing indices were calculated with *PLATON* (Spek, 2020 ▸). For the latter, the H-atom positions in **3**-I and **3**-II were normalized to make the *X*—H distances equal to the average neutron diffraction values (C—H = 1.089 Å and N—H = 1.015 Å) (Allen & Bruno, 2010 ▸), using *Mercury*. Crystal data, data collection and structure refinement details are summarized in Table 1 ▸.

### Computational methods

Density functional theory (DFT) calculations were per­formed using *ORCA* (Version 5.0; Neese *et al.*, 2020 ▸) with a B3LYP/G (VWN5) hybrid functional (20% HF exchange) (Becke, 1993 ▸; Lee *et al.*, 1988 ▸) using a def2-TZVPP basis set (Weigend & Ahlrichs, 2005 ▸) with an auxiliary def2/J basis (Weigend, 2006 ▸). Optimization of the structure used the BFGS method from an initial Hessian according to Almlöf’s model with a very tight self-consistent field convergence threshold (Häser & Almlöf, 1992 ▸). Calculations were made on the free mol­ecule of **3**. The input structure was taken from the crystal structure of **3**-I. The optimized local minimum-energy structure exhibited only positive modes. Cartesian coordinates of the DFT-optimized structure of **3** can be found in the supporting information.

## Results and discussion

Two polymorphic forms of **3** were found to crystallize concomitantly from a solution in aceto­nitrile under ambient conditions, which could be readily distinguished from one another by their external shapes. Colourless block-shaped crystals belong to a monoclinic phase (hereafter **3**-I) and colourless needle-shaped crystals correspond to a triclinic phase, in which twinning by pseudomerohedry was encountered (hereafter **3**-II).

### Mol­ecular structures of 3 in polymorphs I and II

**Figure 2:**
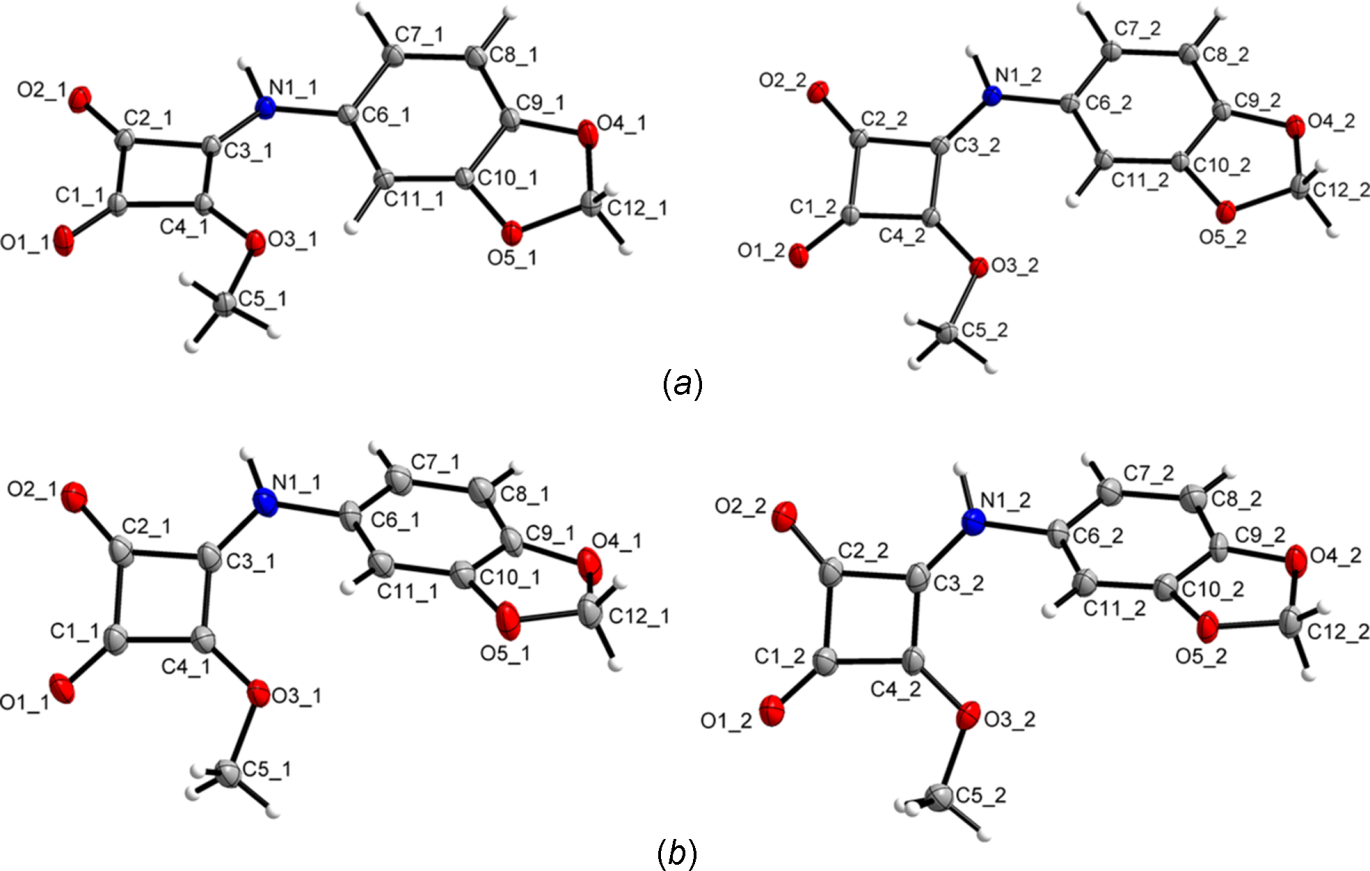
The mol­ecular structures of the crystallographically unique mol­ecules in (*a*) **3**-I and (*b*) **3**-II. The numbers after the underscore indicate crystallographically unique mol­ecules 1 and 2. Displacement ellipsoids are drawn at the 50% probability level. H atoms are represented by small spheres of arbitrary radius.

In both polymeric forms, compound **3** crystallizes with two mol­ecules in the asymmetric unit (*Z*′ = 2). Fig. 2 ▸ depicts displacement ellipsoid plots for both crystallographically unique mol­ecules in each crystal form. In each case, the mol­ecules essentially exhibit the conformation shown in Fig. 1 ▸(*d*), albeit with some tilt between the squaramide and the benzo-1,3-dioxole moieties. In **3**-I, the angle between the mean planes through the four-membered squaramide ring and the six-membered arene ring is 13.5° for mol­ecule 1 and 14.6° in mol­ecule 2. The tilt is significantly larger in **3**-II, as indicated by the angles between the aforementioned mean planes of 41.5° in mol­ecule 1 and 49.4° in mol­ecule 2. The C3—N1—C6—C11 torsion angles also reflect the difference in the mol­ecular conformations in **3**-I and **3**-II (Table 2 ▸).

To evaluate the impact of the overall crystal packing on the conformation of **3**, we performed DFT calculations on the isolated molecule. The resulting minimum energy molecular structure adopts a nearly planar conformation (see supporting information), as revealed by an angle between the mean planes through the four-membered ring and the benzene ring of 5.2° and a C3—N1—C6—C11 torsion angle of −4.7°. It is worth noting that the related 3-methoxy-4-(naph­tha­len-2-yl­am­ino)cyclo­but-3-ene-1,2-di­one adopts approximately the same nearly planar conformation in the crystal (CSD refcode YOHROF; Ávila-Costa *et al.*, 2019 ▸).

### Crystal structure of the monoclinic form **3**-I

**Figure 3:**
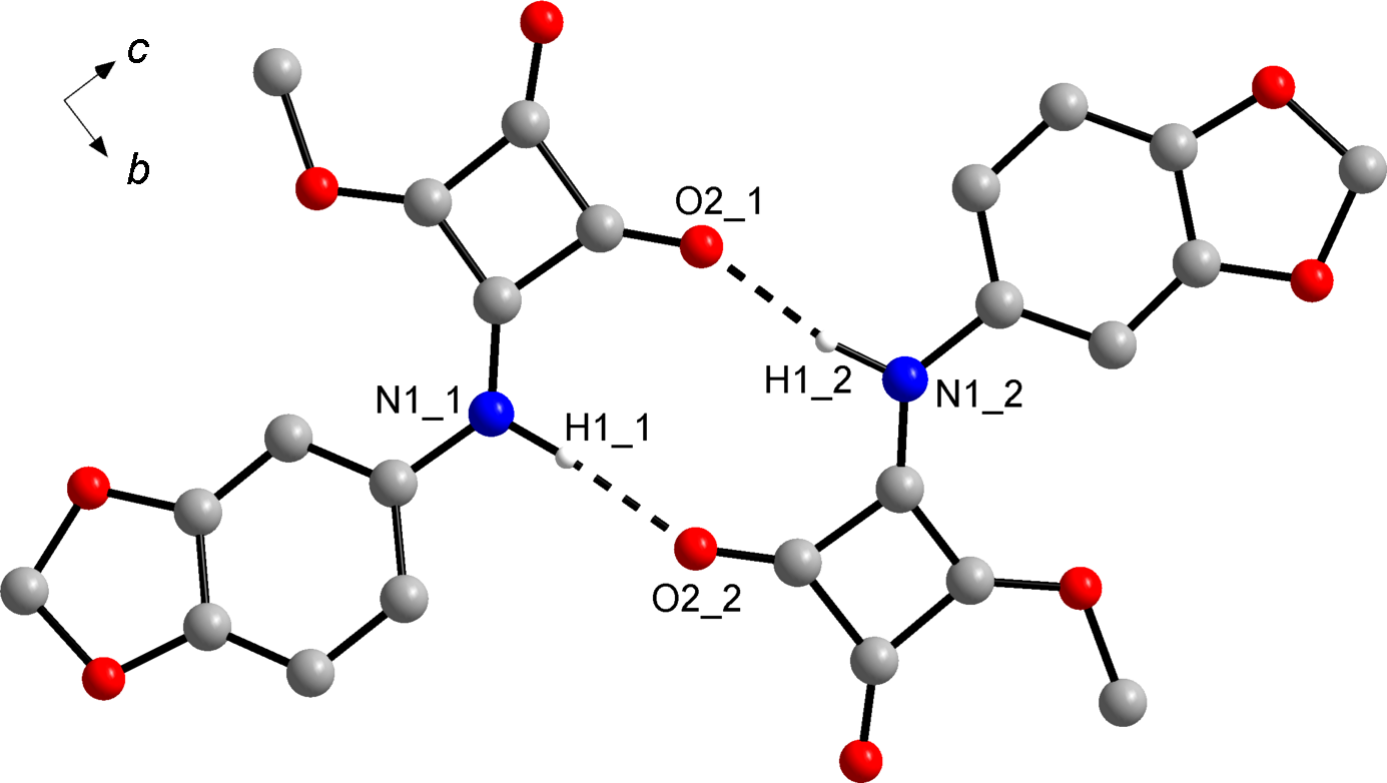
Hydrogen-bond dimer in the crystal structure of **3**-I. Dashed lines represent hydrogen bonds. The numbers after the underscore indicate crystallographically unique mol­ecule 1 and 2. Carbon-bound H atoms have been omitted for clarity.

**Figure 4:**
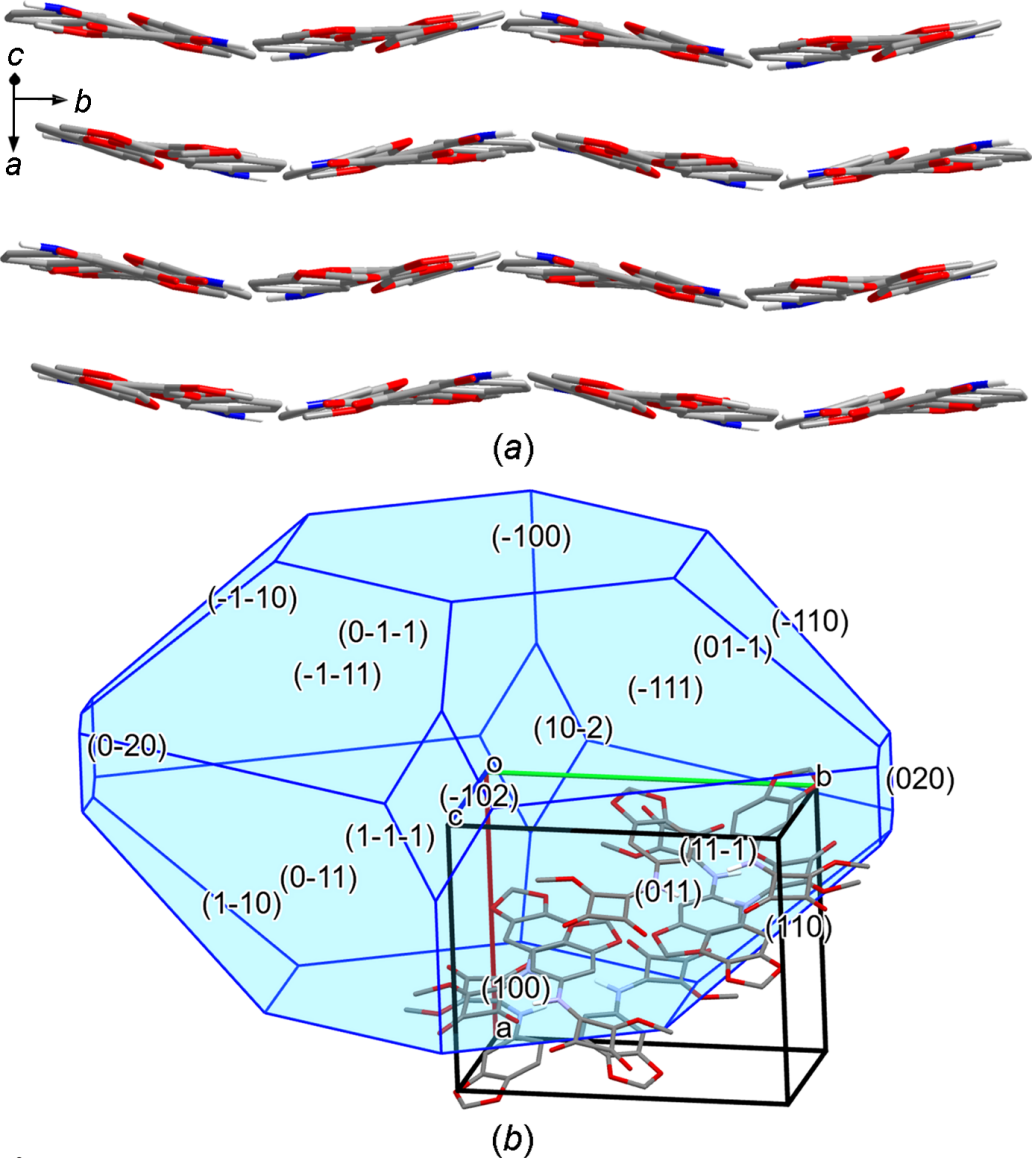
(*a*) Packing diagram of **3**-I, viewed along the [102] direction. (*b*) BFDH morphology calculated for **3**-I. Carbon-bound H atoms have been omitted for clarity.

In the chosen asymmetric unit, the two crystallographically unique mol­ecules in **3**-I form dimers through N—H⋯O=C hydrogen bonds between the amide group and the carbonyl group of an adjacent mol­ecule (Fig. 3 ▸), similar to the above-mentioned YOHROF. The graph-set descriptor is R
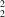
(10) (Bernstein *et al.*, 1995 ▸). Table 3 ▸ lists the corresponding hydrogen-bond parameters. Although the hydrogen-bond dimers so formed lack crystallographic symmetry and their structure also markedly deviates from approximate local *C*_*i*_ symmetry, it is inter­esting to note that the two unique mol­ecules that form a dimer represent enanti­omeric conformers, as indicated by the signs of the C3—N1—C6—C11 torsion angles (Table 2 ▸). The crystal packing in **3**-I is remarkably dense, as revealed by a calculated packing index of 76.4% (Kitajgorodskij, 1973 ▸) and the calculated crystal density (Table 1 ▸). The hydrogen-bond dimers form stacks to give corrugated sheets in the crystal, as revealed by a view along the [102] direction [Fig. 4 ▸(*a*)]. The most prominent feature is stacking of the arene ring of unique mol­ecule 1 and the squaramide ester moiety of unique mol­ecule 2 in adjacent sheets. The distance between the corresponding ring centroids is 3.31 Å. The BFDH morphology calculation, as shown in Fig. 4 ▸(*b*), predicts the shape of the crystals (see supporting information) roughly correctly.

### Crystal structure of the triclinic form **3**-II

**Figure 5:**
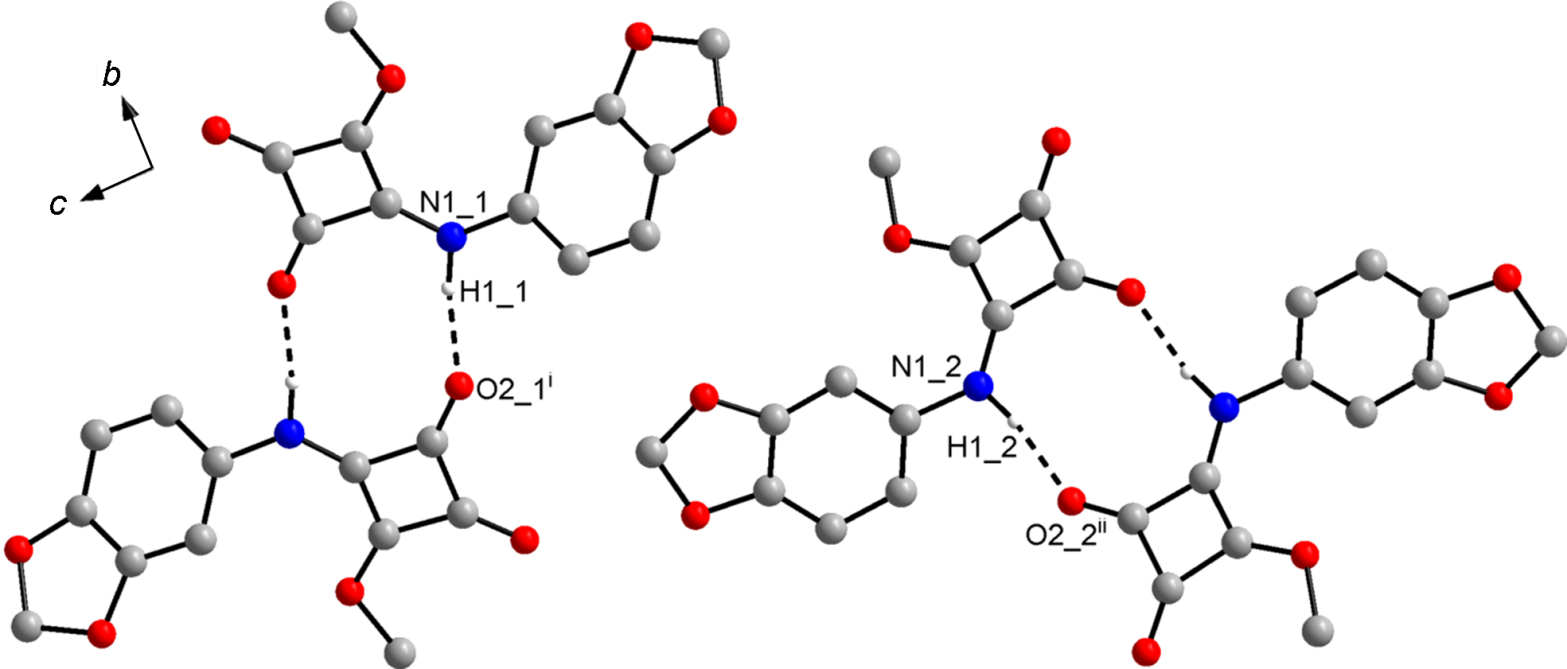
Hydrogen-bond dimers in the crystal structure of **3**-II. Dashed lines represent hydrogen bonds. The numbers after the underscore indicate crystallographically unique mol­ecules 1 and 2. Carbon-bound H atoms have been omitted for clarity. [Symmetry codes: (i) −*x*, −*y* + 1, −*z* + 1; (ii) −*x* + 1, −*y*, −*z*.]

**Figure 6:**
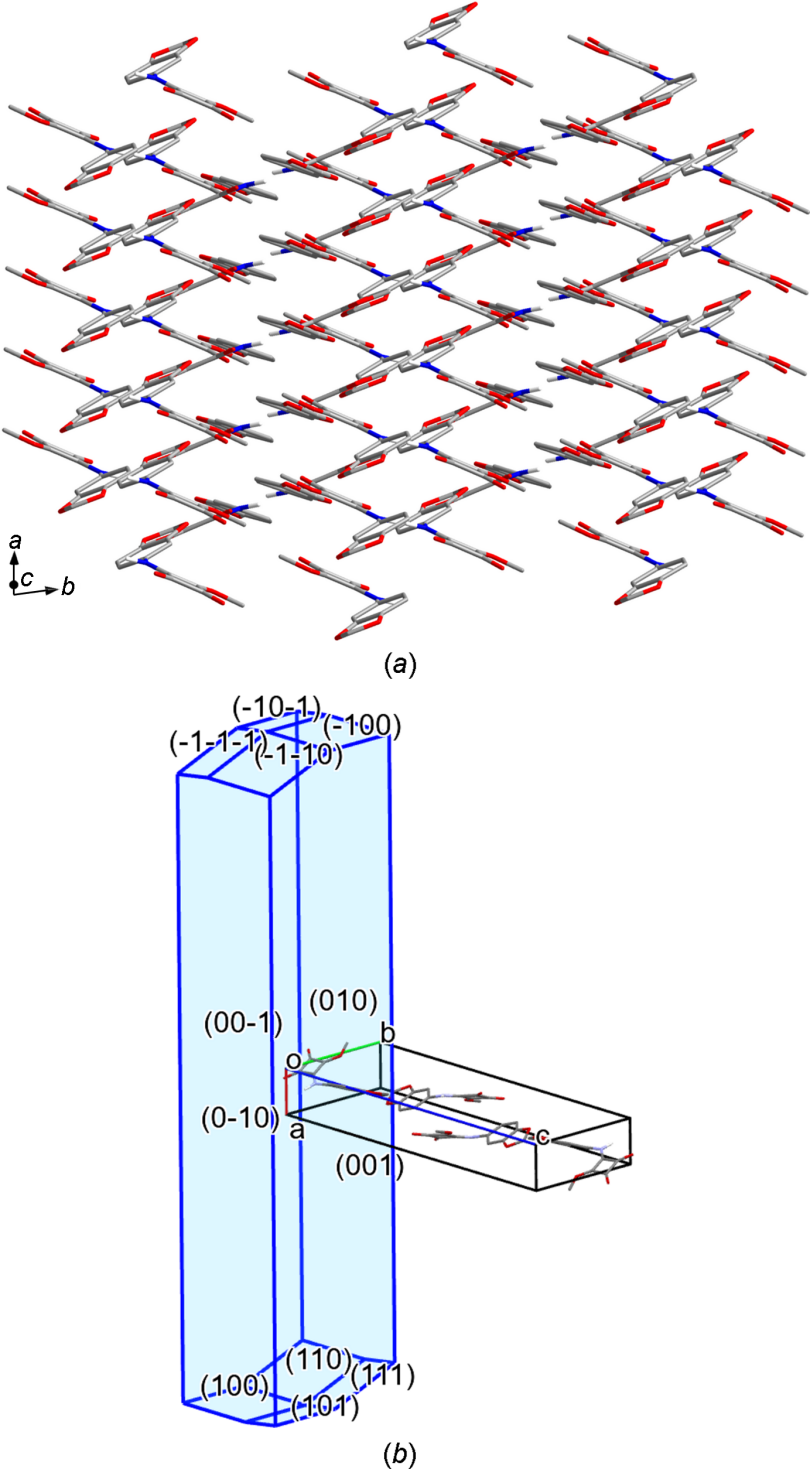
(*a*) Packing diagram of **3**-II, viewed along the [20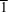] direction. (*b*) BFDH morphology calculated for **3**-II. Carbon-bound H atoms have been omitted for clarity.

The crystal structure of the triclinic polymorph **3**-II likewise features dimers formed through N—H⋯O=C hydrogen bonds with an R
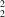
(10) motif (Fig. 5 ▸). Table 4 ▸ lists the associated hydrogen-bond parameters. In contrast to **3**-I, the hydrogen-bond dimers are not formed by crystallographically distinct mol­ecules, but each of the two unique mol­ecules forms a dimer about a crystallographic inversion centre with a symmetry-related mol­ecule (Fig. 5 ▸). The calculated crystallographic den­sity of **3**-II is virtually equal to that of the monoclinic phase **3**-I (Table 1 ▸). Likewise, the packing index calculated for **3**-II at 76.7% is nearly the same as that of **3**-I. In contrast to **3**-I, the arene rings and the squaramide moieties of adjacent mol­ecules each assemble to form stacks. The distances between the ring mean planes are *ca* 3.3 Å. The centroid–centroid separation is 3.70 Å in each case (corresponding to the *a* lat­tice parameter). The overall crystal packing of **3**-II is distinctly different from that of **3**-I. As shown in Fig. 6 ▸(*a*), a view along the [20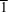] direction reveals a herringbone-like pattern. As for **3**-I, the BFDH morphology calculation predicts the needle shape of the crystals of **3**-II roughly correctly, with the *a* axis representing the needle axis [Fig. 6 ▸(*b*)].

**Figure 7:**
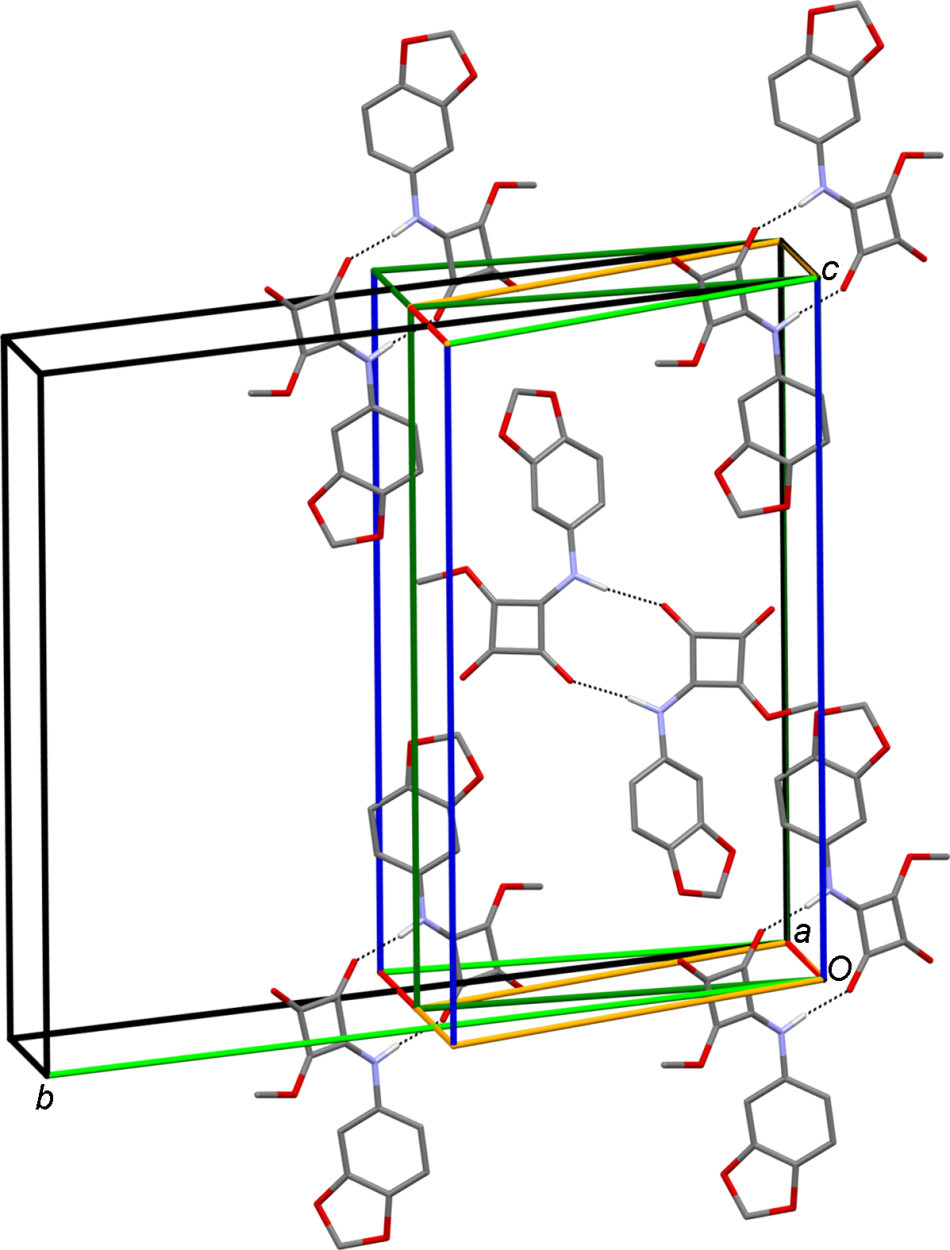
Part of the crystal structure of **3**-II (mol­ecules in the major twin com­ponent) and the relationship between the pseudo-monoclinic *C*-centred unit cell (black line) and the two twin components with respect to the triclinic primitive cell (dark-green and orange lines). Dashed lines represent hydrogen bonds. Carbon-bound H atoms have been omitted for clarity.

The crystals of **3**-II were twinned by pseudomerohedry (Parkin, 2021 ▸; Parsons, 2003 ▸). The conventional triclinic primi­tive cell of **3**-II can be transformed to a *C*-centred cell as follows:
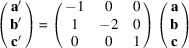


The *C*-centred cell so obtained simulates monoclinic metrics with *a*′ = 3.700, *b*′ = 24.640, *c*′ = 22.846 Å and β′ = 93.03°. The twin operation in the nonstandard space group setting *C*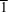 is a twofold rotation about the *b*-axis direction:
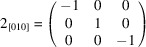


A mirror operation about the plane perpendicular to the *b*-axis direction of the *C*-centred cell is an equal description of the twinning. The second twin component relative to the reduced cell can be derived from:
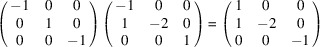


The twin operation expressed with respect to the reduced cell can then be calculated as follows:
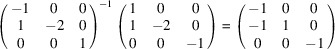


In the triclinic axis system of the reduced cell, this represents a twofold rotation about the [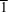
20] direction. Fig. 7 ▸ shows the relationship between the pseudo-monoclinic *C*-centred unit cell and the two twin components with respect to the primitive triclinic cell. The ratio of the fractional volume con­tributions of the two twin components refined to 0.584 (2):0.416 (2). A similar case of twinning by pseudomerohedry of a triclinic crystal of an organic compound was reported by Bolte & Kettner (1998 ▸).

### Anti­mycobacterial evaluation

We wondered whether compound **3** as a precursor to anti­mycobacterial squaramides (Li *et al.*, 2020 ▸) might itself exhibit anti­mycobacterial activity. Therefore, we evaluated its activity against the NTM species *Mycobacterium smegmatis* and *Mycobacterium abscessus* subsp. *abscessus. M. smegmatis* is a generally considered non-pathogenic model organism in early-stage anti-TB drug discovery (Sundarsingh *et al.*, 2020 ▸), whereas *M. abscessus* is an opportunistic pathogen, which can cause difficult-to-treat lung disease resembling pulmonary TB and extrapulmonary infections in susceptible hosts (Abdelaal *et al.*, 2022 ▸). We performed susceptibility testing against *M. smegmatis* mc^2^ 155 pTEC27 and *M. abscessus* ATCC 19977 pTEC27 (expressing tomato red fluorescent protein) using the broth microdilution method (Middlebrook 7H9 medium sup­plemented with 10% albumin–dextrose–saline and containing 0.05% polysorbate 80) with optical density and fluorescence based readout, as described previously (Lang *et al.*, 2023 ▸). Up to a compound concentration of 100 µM, however, no growth inhibition of the two aforementioned mycobacterial strains was observed. The results appear to be in line with the SAR studies reported by Tantry *et al.* (2017 ▸) and Li *et al.* (2020 ▸), which found that the 2-picolyl group is critical for activity against *M. tuberculosis* H37Rv.

## Conclusions

We report two concomitant polymorphs of the title compound **3** and structurally characterized them by X-ray crystallog­raphy. Both the monoclinic form **3**-I and the triclinic form **3**-II were found to crystallize with two mol­ecules in the asymmetric unit (*Z*′ = 2). The mol­ecular conformations differ significantly between the two polymorphs and variously differ depending on the polymorph. DFT calculations on the isolated mol­ecule suggest that a planar conformation is preferred. Whereas the packing of the mol­ecules in **3**-I is characterized by alternate stacking of arene rings and squaramide ester moieties of adjacent mol­ecules, in **3**-II, these groups each assemble to form columns. Crystallographic densities and packing indices calculated for **3**-I and **3**-II indicate that the crystal packing is equally dense within experimental error, which suggests that the difference in energy between the two polymorphs is small. This possibly explains why concomitant crystallization of both crystal forms occurred. As expected, and consistent with previous SAR studies, no *in vitro* activity of **3** against two mycobacterial strains was observed.

## Supplementary Material

Crystal structure: contains datablock(s) 3-I, 3-II, global. DOI: 10.1107/S2053229624006211/vp3038sup1.cif

Structure factors: contains datablock(s) 3-I. DOI: 10.1107/S2053229624006211/vp30383-Isup2.hkl

Supporting information file. DOI: 10.1107/S2053229624006211/vp30383-Isup4.cdx

Structure factors: contains datablock(s) 3-II. DOI: 10.1107/S2053229624006211/vp30383-IIsup3.hkl

Supporting information file. DOI: 10.1107/S2053229624006211/vp30383-IIsup5.cdx

Supporting information file. DOI: 10.1107/S2053229624006211/vp30383-Isup6.cml

Supporting information file. DOI: 10.1107/S2053229624006211/vp3038sup8.pdf

Supporting information file. DOI: 10.1107/S2053229624006211/vp3038sup9.txt

CCDC references: 2365204, 2365205

## Figures and Tables

**Table 1:** Experimental details For both structures: C_12_H_9_NO_5_. Experiments were carried out at 100 K with Mo *K*α radiation using a Bruker D8 Venture diffractometer. The absorption correction was Gaussian (*SADABS*; Bruker, 2016 ▸).

	**3**-I	**3**-II
Crystal data		
*M*_r_	247.20	247.20
Crystal system, space group	Monoclinic, *P*2_1_/*c*	Triclinic, *P* 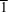
*a*, *b*, *c* (Å)	13.0541 (7), 13.4304 (7), 13.1257 (7)	3.7001 (4), 12.4583 (15), 22.846 (3)
α, β, γ (°)	90, 115.354 (2), 90	89.550 (8), 86.967 (6), 81.460 (6)
*V* (Å^3^)	2079.57 (19)	1040.0 (2)
*Z*	8	4
μ (mm^−1^)	0.13	0.13
Crystal size (mm)	0.07 × 0.07 × 0.05	0.12 × 0.05 × 0.03

Data collection		
*T*_min_, *T*_max_	0.992, 0.997	0.991, 0.998
No. of measured, independent and observed reflections	806109, 6382, 5025 [*I* ≥ 2σ(*I*)]	76269, 5138, 3902 [*I* > 2σ(*I*)]
*R*_int_	0.148	0.134
(sin θ/λ)_max_ (Å^−1^)	0.717	0.668

Refinement		
*R*[*F*^2^ > 2σ(*F*^2^)], *wR*(*F*^2^), *S*	0.030, 0.080, 1.11	0.064, 0.170, 1.04
No. of reflections	6382	5138
No. of parameters	397	337
No. of restraints	0	1
H-atom treatment	All H-atom parameters refined	H atoms treated by a mixture of independent and constrained refinement
Δρ_max_, Δρ_min_ (e Å^−3^)	0.24, −0.24	0.38, −0.36

Computer programs: *APEX5* (Bruker, 2022 ▸), *SAINT* (Bruker, 2019 ▸), *SHELXT* (Sheldrick, 2015*a* ▸), *olex2.refine* (Bourhis *et al.*, 2015 ▸), *SHELXL* (Sheldrick, 2015*b* ▸), *DIAMOND* (Brandenburg, 2018 ▸), *Mercury* (Macrae *et al.*, 2020 ▸) and *publCIF* (Westrip, 2010 ▸).

**Table 2:** Selected torsion angles (°) for **3**-I and **3**-II.

	**3**-I	**3**-II
C3_1—N1_1—C6_1—C11_1	15.64	−42.0 (5)
C3_2—N1_2—C6_2—C11_2	−18.46 (11)	−49.3 (5)

**Table 3:** Hydrogen-bond geometry (Å, °) for **3**-I

*D*—H⋯*A*	*D*—H	H⋯*A*	*D*⋯*A*	*D*—H⋯*A*
N1_1—H1_1⋯O2_2	1.037 (15)	1.864 (15)	2.8963 (10)	172.9 (12)
N1_2—H1_2⋯O2_1	1.026 (15)	1.901 (15)	2.8772 (10)	157.8 (12)

**Table 4:** Hydrogen-bond geometry (Å, °) for **3**-II

*D*—H⋯*A*	*D*—H	H⋯*A*	*D*⋯*A*	*D*—H⋯*A*
N1_1—H1_1⋯O2_1^i^	0.94 (3)	1.95 (4)	2.880 (4)	168 (4)
N1_2—H1_2⋯O2_2^ii^	0.94 (3)	1.91 (3)	2.831 (4)	167 (4)

Symmetry codes: (i) 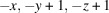; (ii) 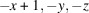.
