# Supplementary material for: 3-[(Benzo-1,3-dioxol-5-yl)amino]-4-meth­oxy­cyclo­but-3-ene-1,2-dione: polymorphism and twinning of a precursor to an anti­mycobacterial squaramide
Source: Acta Crystallogr C Struct Chem. 2024 Jul 5;80(Pt 8):375–82. doi: 10.1107/S2053229624006211 (PMC11299207; doi:10.1107/S2053229624006211)
Supplement: Supplementary file 7 [file c-80-00375-sup8.pdf]

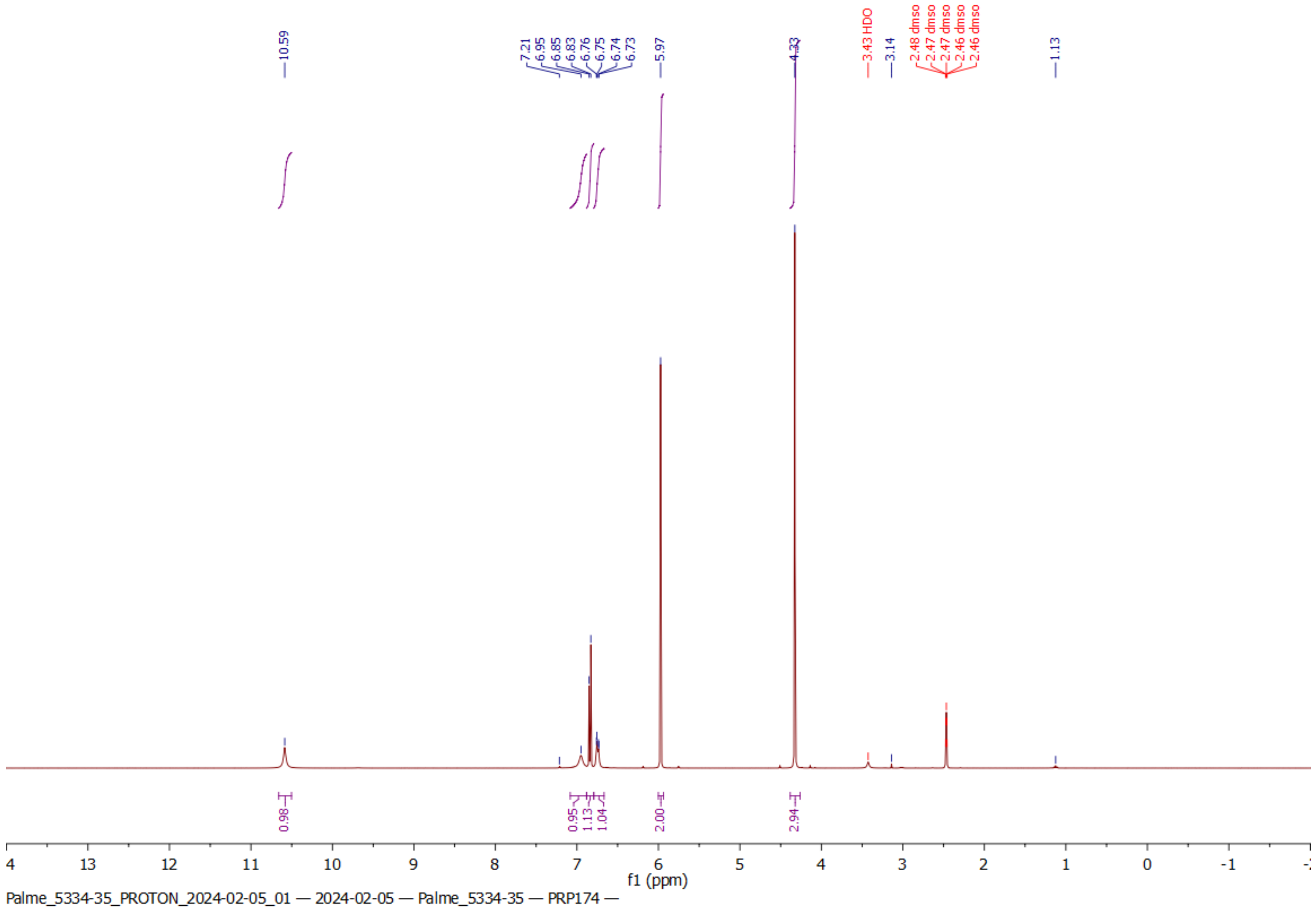

Figure S1 ^{1}H NMR spectrum of compound **3** in DMSO-*d*6.

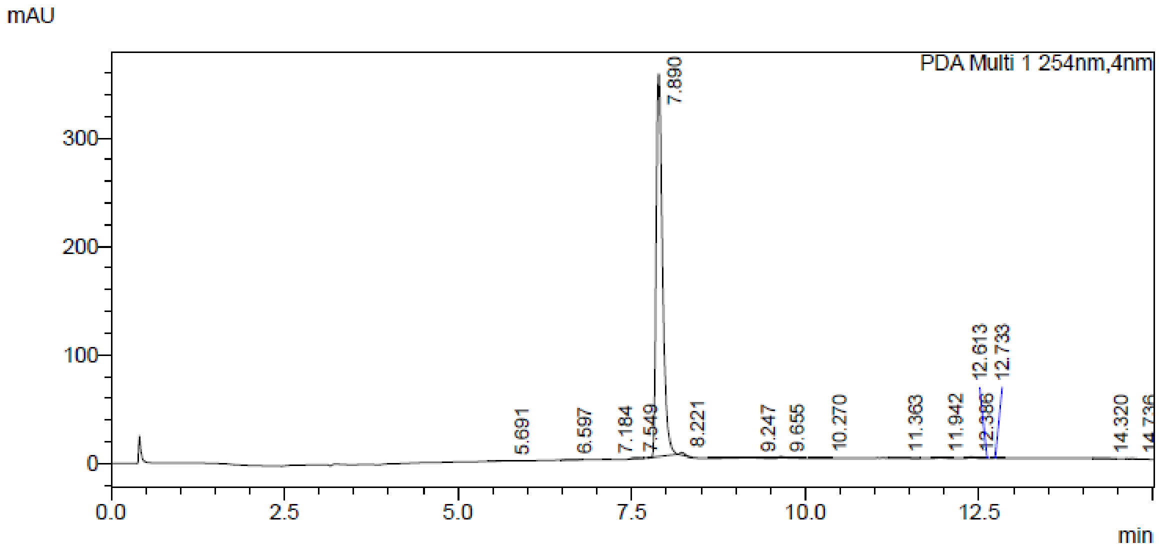

Figure S2 RP-HPLC chromatogram of compound **3** (254 nm detection).

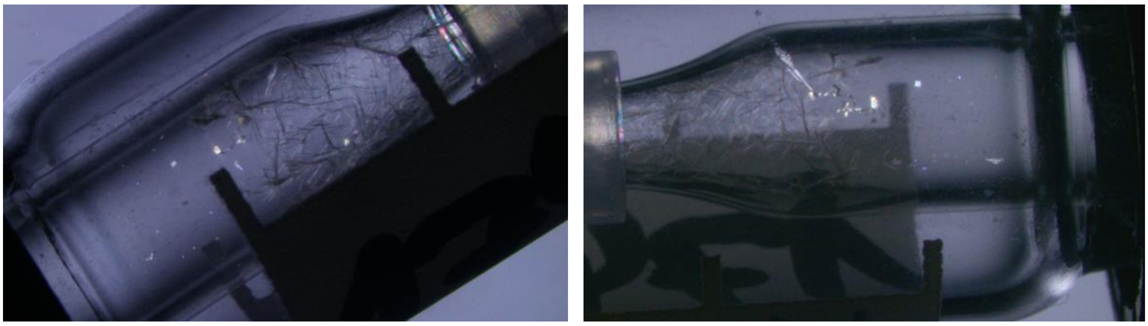

Figure S3 Microscope images of concomitantly crystallized block-shaped crystals of **3**-I and needle-shaped crystals of **3**-II from a solution in acetonitrile.

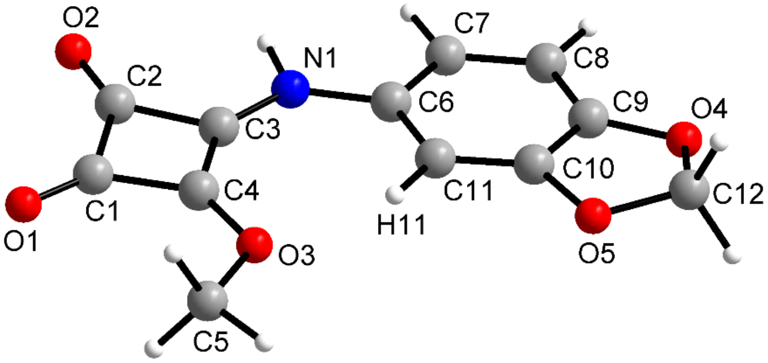

Figure S4 DFT-optimized structure of the free molecule of **3**.

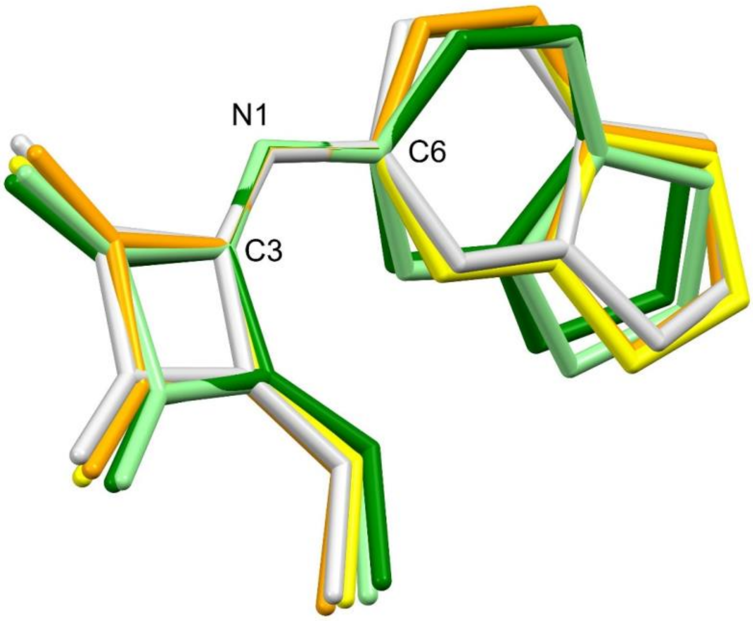

Figure S5 Structure overlay plot of the molecular structures of both crystallographically unique molecules in **3**-I (molecule 1: yellow; molecule 2: orange) and **3**-II (molecule 1: light green; molecule 2: dark green) and the DFT-optimized structure of the free molecule of **3** (grey). The molecular structures were superimposed at C3, N1 and C6 (C3–N1–C6–C11 torsion angles positive). Hydrogen atoms are omitted for clarity.
